# Reaction of 2,2,4,4-tetrakis(trifluoromethyl)-1,3-dithiethane with *N*-vinyl compounds

**DOI:** 10.3762/bjoc.9.295

**Published:** 2013-11-21

**Authors:** Viacheslav A Petrov, Will Marshall

**Affiliations:** 1 DuPont Central Research and Development, Experimental Station, PO Box 80500, Wilmington DE 19880-0500, USA; 2 DuPont Corporate Center for Analytical Sciences, Experimental Station, PO Box 80500, Wilmington DE 19880-0500, USA

**Keywords:** 1-(hexafluoroisopropyl)-3-vinyl-1,3-dihydro-2*H*-imidazole-2-thione, 2,2,4,4-terakis(trifluoromethyl)-1,3-dithiethane, 4-amino-2,2-bis(trifluoromethyl)thietanes, organo-fluorine

## Abstract

The reaction of hexafluorothioacetone dimer (2,2,4,4-tetrakis(trifluoromethyl)-1,3-dithiethane, **1**) with vinylamides leads to the rapid formation of [2 + 2] cycloadducts: 4-amino-2,2-bis(trifluoromethyl)thietanes. The reaction proceeds in polar solvents (DMF, DMSO) in the absence of a catalyst at elevated temperature producing the corresponding cycloadducts in 47–86% yield. The reaction of *N*-vinylimidazole unexpectedly led to the formation of the corresponding 1-(hexafluoroisopropyl)-3-vinyl-1,3-dihydro-2*H*-imidazole-2-thione (**5**). The structure of this compound, along with the structures of two new thietanes was confirmed by single crystal X-ray diffraction.

## Introduction

Hexafluorothioacetone (HFTA) and hexafluorothioacetone dimer (2,2,4,4-tetrakis(trifluoromethyl)-1,3-dithiethane (**1**) were shown to react with a wide variety of electron-rich olefins and dienes, producing the corresponding cycloadducts. Reported examples include the corresponding thietanes, derived from the reaction of HFTA with vinyl ethers, vinyl sulfides, cyclic dienes and styrenes [1–2], fluoride anion-catalyzed reactions of compound **1** with vinyl ethers [1,3–4], vinyl sulfides [3], ketene dimethylacetal [5], styrenes [6–7], cyclic dienes [8] and quadricyclane [9]. At this point, no data for the reaction of HFTA or HFTA dimer with vinylamines can be found in the literature, despite the fact that (CF_3_)_2_C=C=S (which exhibits a reactivity very similar to the reactivity of HFTA) was reported to react with *N*-vinylcarbazole to give a [2 + 2] cycloadduct [10]. In this paper we report the synthesis of 2,2-bis(trifluoromethyl)-4-amino-substituted thietanes by a reaction of **1** with selected vinylated amino compounds.

## Results and Discussion

**Scheme 1:**
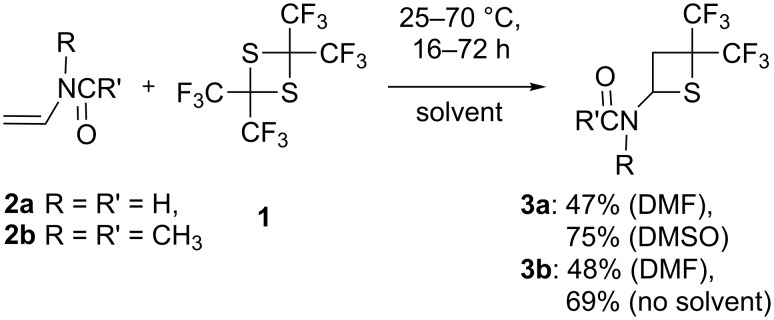
Reaction of vinylamides **2a**,**b** with **1**.

It was found that *N*-vinylamides **2a**,**b** react with **1** in the absence of a catalyst in polar solvents to produce the corresponding thietanes **3a**,**b** in moderate yield (Scheme 1). The reaction can be carried out at either ambient or elevated temperature (see Table 1).

**Table 1:** Reaction conditions, yields, melting/boiling points and MS data for compounds **3a–e** and **5**.

Compound	Method^a^	Temp. (°C)/Time (h), Solvent	Yield (%)	Mp (°C), (bp °C/mmHg)	MS (*m*/*z*)

**3a**	A A	25/72, DMF 25/16, DMSO	47 75^c^	orange oil^b^	253 (M^+^, C_6_H_5_F_6_NOS^+^)
**3b**	A A B	70/14, DMF 70/16, neat^d^ 25–50/12, DMF	48 69 56	93–94	281 (M^+^, C_8_H_9_F_6_NOS^+^)
**3c**	A	70–80/2, DMF	86	81–82	293 (M^+^, C_9_H_9_F_6_NOS^+^)
**3d**	A	70/24, DMF	55–86	80–81	321 (M^+^, C_11_H_13_F_6_NOS^+^)
**3e**	A B	70/2, DMF 25/16, DMF	55 60	103–103.5	375 (C_17_H_11_F_6_NS^+^)
**5**	A A	25/48, DMF 70/2, DMF	50 48	40–41 (74/2)	276 (M^+^, C_8_H_6_F_6_N_2_S^+^)

^a^Method A: no catalyst; method B: HFP, S_x_, DMF, one-pot reaction. ^b^Purity 98%, GC–MS, NMR. ^c^90% by NMR. ^d^Three molar excess of vinylamide was used.

**Figure 1:**
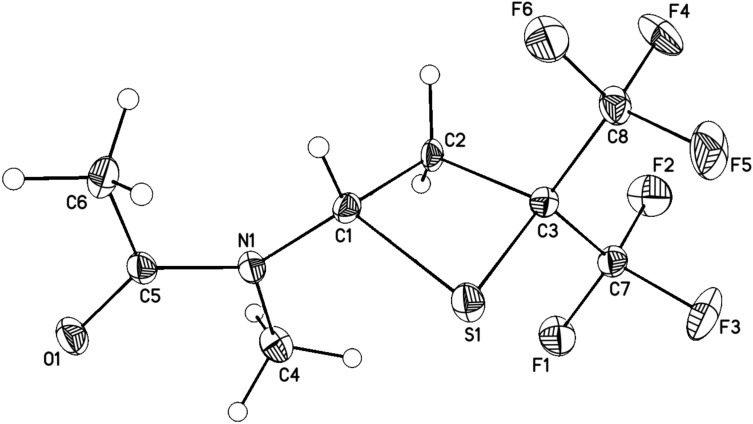
Crystal structure of **3b** with thermal ellipsoids drawn at the 30% probability level.

It should be pointed out that in case of compound **2b** the reaction can be performed in an excess of vinylamine as the solvent. The reaction is highly selective (NMR), leading to the exclusive formation of thietanes and fluctuations in the isolated yields of compounds **3a** and **3b** are connected to the isolation protocol (see Experimental). In contrast to the corresponding cycloadducts of vinyl ethers and HFTA (2,2-bis(trifluoromethyl)-4-alkoxythietanes), which are high-boiling liquids [3], thietanes **3a** and **3b** are solids at room temperature. The structure of purified **3b** was established by single crystal X-ray diffraction (Figure 1).

**Scheme 2:**
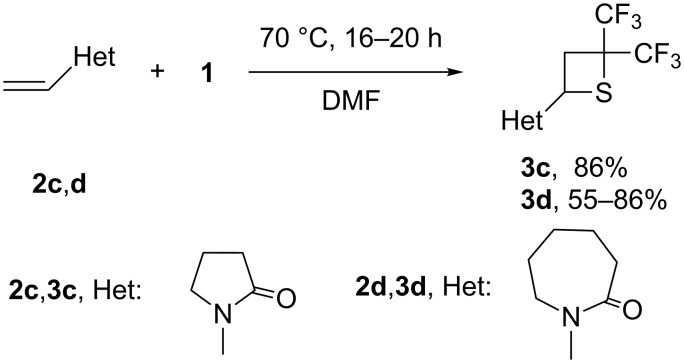
Reaction of *N*-vinyllactames **2c**,**d** with **1**.

Interestingly, the ^19^F and ^1^H NMR spectra of both compounds **3a** and **3b** show two sets of signals, probably being a result of restricted rotation around the C–N bond in the amide fragment, similar to restricted rotation of –N(CH_3_)_2_ in dimethylformamide. Cyclic lactames **2c**,**d** bearing a vinyl group at the nitrogen also undergo reaction with **1** producing the corresponding thietanes **3c**,**d** (Scheme 2).

It should be pointed out that in sharp contrast to compounds **3a**,**b**, NMR spectra of **3c** and **3d** exhibit only one set of signals**,** which might be indicative of a significantly lower rotation barrier around the C–N bond in these two materials. The structures of both **3c** and **3d** were established by single crystal X-ray diffraction. Compounds **3b** and **3e** were also prepared in 56–60% yield by using a one-step process in which dimer **1** was generated by the reaction of hexafluoropropene (HFP) with sulfur in the presence of CsF as catalyst, followed by the addition of vinyl compounds **2b** or **2e** without the isolation of dimer **1**.

**Scheme 3:**
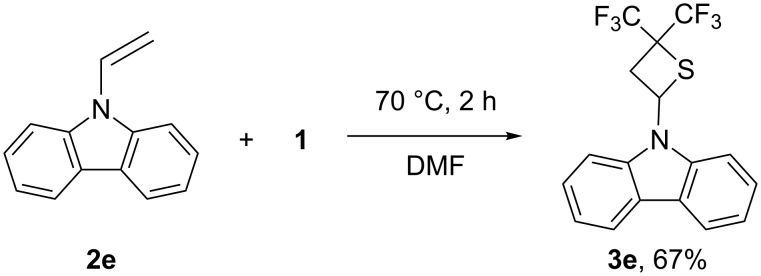
Reaction of *N*-vinylcarbazole with **1**.

**Figure 2:**
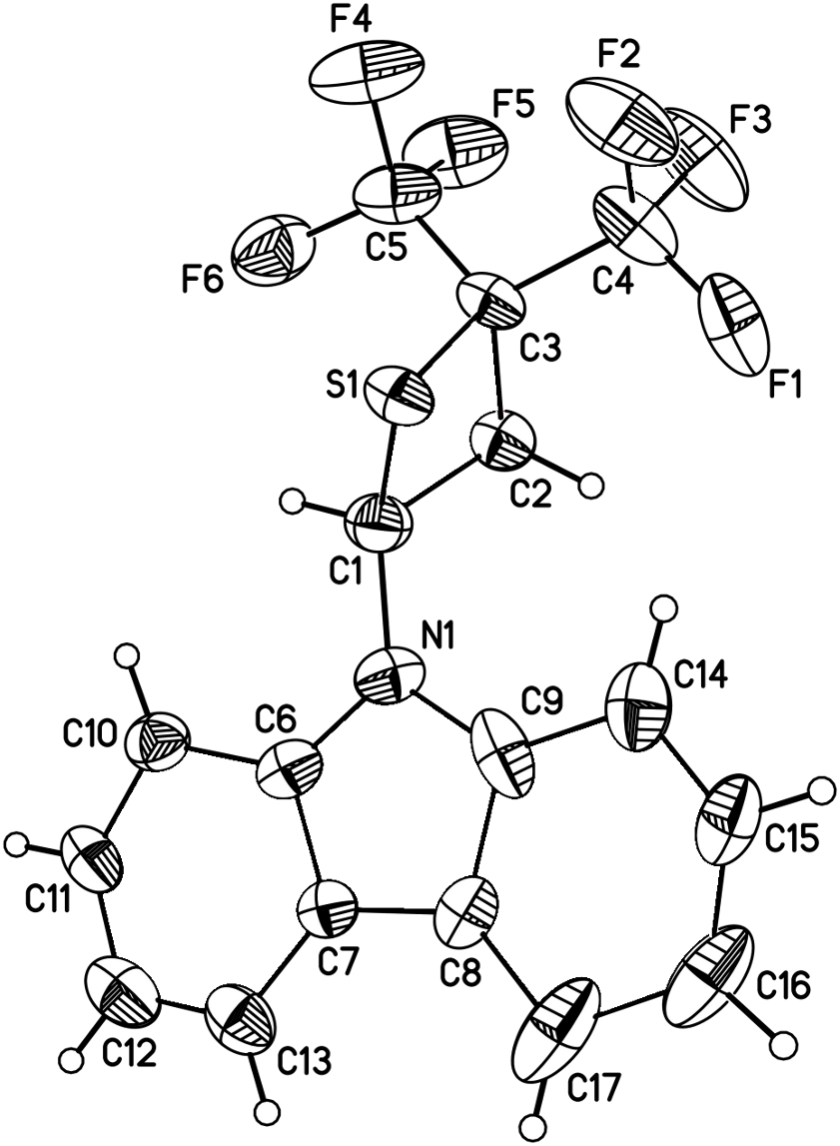
Crystal structure of **3e** with thermal ellipsoids drawn at the 30% probability level. Disordered atoms are omitted for clarity.

*N*-vinylcarbazole (**2e**) was found to have the highest reactivity towards **1**. Indeed, when the mixture of **2e** and **1** in DMF was heated at 70 °C, no starting carbazole was found in the reaction mixture after 2 hours (NMR). This reaction led to the selective formation of thietane **3e**, isolated in 67% yield (Scheme 3). The structure of **3e** was established by single crystal X-ray diffraction (Figure 2).

Interestingly, the ^19^F and ^1^H NMR spectra of **3e** showed only one set of signals, despite the fact that in the corresponding cyclopropane (prepared by the reaction of compound **3e** with PBu_3_), restricted rotation around the C–N bond was observed [11].

**Scheme 4:**
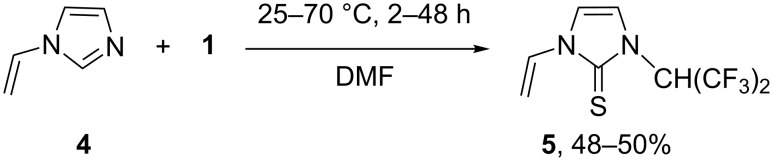
Formation of thione **5** in reaction of **4** and **1**.

**Figure 3:**
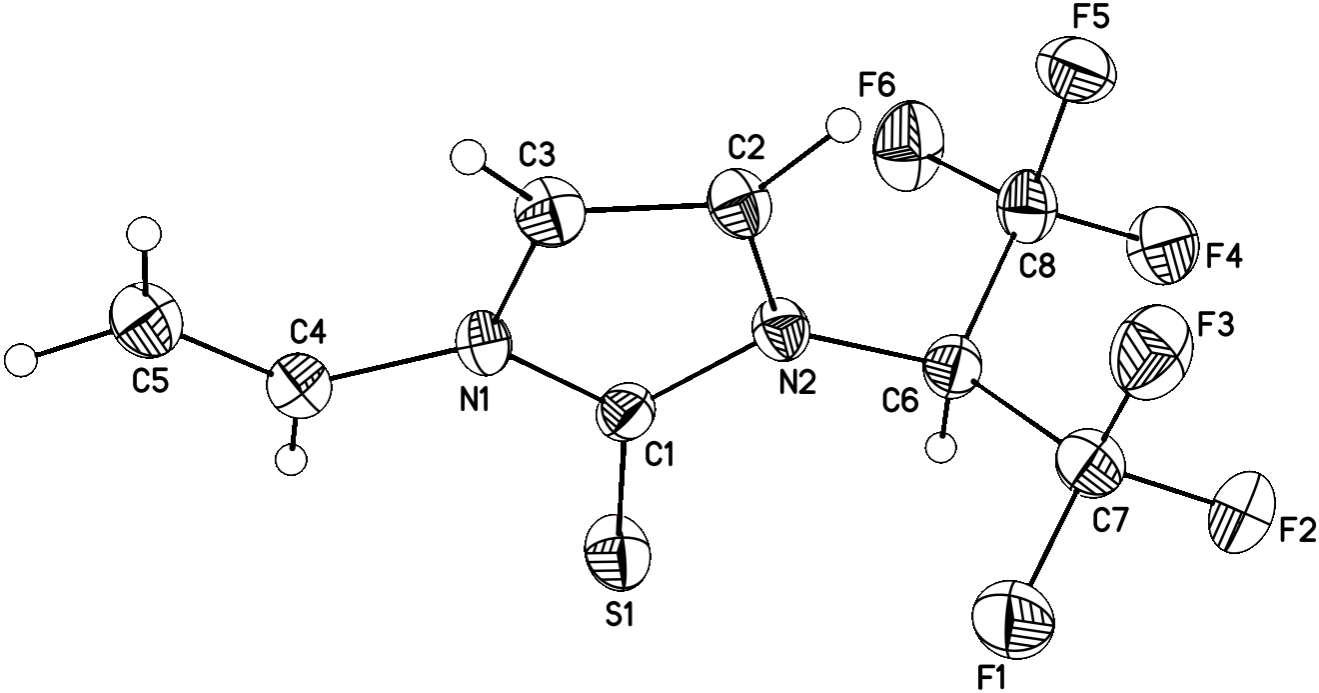
Crystal structure of **5** with thermal ellipsoids drawn at the 30% probability level.

*N*-vinylimidazole (**4**) was found to have a different reactivity profile. The reaction of **4** with **1** (25 °C, 48 h) unexpectedly led to the formation of thione **5** (rather than the corresponding thietane) as a sole product (NMR, Scheme 4). Thione **5** was isolated in 48–50% yield and its structure was established by single crystal X-ray diffraction (Figure 3).

At elevated temperature, the reaction of **4** with **1** was significantly faster and it was completed after 2 h at 70 °C in DMF. On the other hand, *N*-vinyl-1,3,4-triazole was found to be inert towards dimer **1** and even after prolonged heating (70 °C, 48 h, DMF) only starting materials were observed in the reaction mixture (NMR).

The mechanistic aspect of thione **5** formation is not clear at this point. The formation of the corresponding thiones in the reaction of **1** with *N*-alkylimidazoles was found to be common in nature.

## Experimental

^1^H NMR and ^19^F NMR spectra were recorded on a Bruker DRX-500 (499.87 MHz) instrument by using CFCl_3_ or TMS as an internal standard. CDCl_3_ was used as a lock solvent. GC and GC–MS analyses were carried out on a HP-6890 instrument by using an HP FFAP capillary column and either TCD (GC) or mass-selective (GC–MS) detectors, respectively. Dry DMF (water <100 ppm), vinylamines **2a–e** and **4** (Aldrich), sulfur (Alfa Aesar, sublimed, 99.5%, 100 mesh) and hexafluoropropene (DuPont) were purchased and used without further purification. CsF (Aldrich, 99%) was dried at 100–120 °C under dynamic vacuum and stored and handled inside of glove box.

Compound **1** (liquid, purity 93–98%) was prepared by using modified literature procedure reported in [12] and was used without further purification.

Due to the high ratio of sulfur to fluorine, the elemental analysis of new materials was not attempted. The purity of all new compounds established by GC and NMR spectroscopy was at least 98%.

### Crystallography

X-ray data for **3b**, **3e** and **5a** were collected at −100 °C by using a Bruker 1K CCD system equipped with a sealed tube molybdenum source and a graphite monochromator. The structures were solved and refined with the Shelxtl [13] software package, refinement by full-matrix least squares on F^2^, scattering factors from Int. Tab. Vol. C Tables 4.2.6.8 and 6.1.1.4. Crystallographic data (excluding structure factors) for the structures in this paper have been deposited with the Cambridge Crystallographic Data Centre as supplementary publication nos. CCDC #956557-956559. Copies of the data can be obtained, free of charge, on application to CCDC, 12 Union Road, Cambridge CB2 1EZ, UK, (fax: +44 1223 336033 or e-mail: deposit@ccdc.cam.ac.uk).

### Reaction of **1** with **2a–e** and **4** (typical procedure)

Method A: To a solution of the corresponding vinyl compound (0.01–0.02 mol) in 15 mL of solvent was added 0.005–0.01 mol of dimer **1**. The reaction mixture was agitated for 2–72 h at either ambient or elevated temperature. The reaction mixture was diluted by water (300 mL, extracted with hexane (3 × 50 mL). Combined organic layers were washed by water (3 × 200 mL) and dried over MgSO_4_. The solvent was removed under reduced pressure and the residue was purified either by crystallization from hexane or distillation (compound **5**).

Method B: Inside a glove box a dry three-necked round-bottomed flask was charged with 0.5–1.0 g of dry CsF. The flask was taken out and equipped with dry-ice condenser, thermocouple, gas inlet tube and 100 mL of dry DMF was added by using a syringe, followed by the addition of 6.4 g (0.2 mol) of sublimed sulfur. The reaction mixture was agitated for 15–20 min, while the temperature was raised up to 35–40 °C. This process was accompanied by the development of a dark blue–brown color of the reaction mixture. Hexafluoropropene 32 g (0.21 mol) was added as a gas to the reaction mixture, and the temperature usually rose to 65–70 °C during the addition and at the end all sulfur went into solution. The reaction mixture was cooled to an ambient temperature followed by the addition of the corresponding *N*-vinyl compound, and the reaction mixture was agitated for 12–16 h. Afterwards the mixture was diluted with water (500 mL) and extracted with hexane (3 × 100 mL). The combined organic layers were washed with water (3 × 200 mL) and dried over MgSO_4_. The solvent was removed under reduced pressure, and the residue was purified either by crystallization from hexane or distillation (see Table 1 for conditions and yields and Table 2 for NMR data).

**Table 2:** ^1^H, ^19^F, ^13^C NMR data for compounds **3a–e** and **5**.

Entry	Compound	^1^H NMR^a^ (δ, ppm, *J*, Hz)	^19^F NMR^a^ (δ, ppm, *J*, Hz)	^13^C NMR^a,b^ (δ, ppm, *J*, Hz)

1	**3a**^ c^	Major: 3.03 (1H, m), 3.55 (1H, m), 5.82 (1H, dd, 7.8), 6.40 (1H, br.s), 8.18 (1H, s)	Major: −73.80 (s)^d^	Major: 38.4, 41.2, 46.6 (sept., 32.0), 123.3 (q, 278.0), 124.2 (q, 276.0), 161.3
		Minor: 3.21 (1H, dd), 3.51 (1H, dd), 5.35 (1H, m), 6.39 (br. s), 8.13 (1H, d, 11.0)	Minor: −73.65 (m), −73.59 (m)	
2	**3b**^e^	Major: 3.13 (3H, s), 2.19 (3H, s), 3.15 (1H, m), 3.32 (1H, m), 6.67 (1H, t, 7.8)	Major: −72.73 (3F, q, 9.6), −73.78 (3F, q, 9.6)	Major: 22.51, 29.6, 34.5, 45.8 (sept., 31.8), 47.5, 123.5 (q, 280), 124.4 (q, 284), 171.8
		Minor: 2.17 (s), 3.59 (s), 5.89 (m)	Minor: −72.66 (br.s), −73.78	
3	**3c**	2.11 (2H, sept, 7.0), 2.44 (2H, m), 3.25 (1H, dd), 3.37 (1H, dd), 3.60 (1H, m), 3.85 (1H, m), 6.16 (1H, t, 8.0)	−73.09 (3F, q, 9.6), −73.91 (3F, q, 9.6)	17.5, 31.3, 34.5, 41.9, 45.2, 45.8 (sept, 33.1), 123.4 (q, 279), 124.5 (q, 279), 175.6
4	**3d**	1.68 (3H, m), 1.76 (3H, m), 2.56 (1H, t, 8.1), 3.21 (2H, m), 3.70 (2H, m), 6.63 (1H, t, 8.1)	−72.63 (3F, q, 10.3), −73.70 (3F, q, 10.3)	23.23, 29.5, 29.6, 35.7, 37.4, 42.7, 46.0 (sept,. 31.9), 47.4, 123.4 (q, 277), 124.6 (q, 279), 176.5
5	**3e**	3.45 (1H, m), 4.50 (1H, m), 6.75 (1H, t), 7.33 (2H, m), 7.50 (2H, m), 7.54 (2H, m), 7.92 (2H, m)	−70.81 (3F, q, 8.0), −73.22 (3F, q, 8.0)	38.2, 46.9 (sept, 32.0), 48.9, 110.6 (q, 1.5), 120.6, 121.2, 123.5 (q, 281), 124.5, 126.3, 130.3
6	**5**	5.06 (1H, dd, 8.8, 2.0), 5.25 (1H, dd, 16.3, 2.5), 6.72 (sept, 7.1), 6.99 (1H,s), 7.06 (1H, d, 2.5), 7.50 (1H, dd 16.3, 8.8)	−69.19 (d, 7.1)	

^a^In CDCl_3_ as lock solvent. ^b^{H}C^13^ NMR spectra. ^c^Two partially overlapped sets of signals, ratio 5:1. ^d19^F NMR of **3a** in the presence of DMF (CDCl_3_): Major: −73.58 (3F, q, 10.1 Hz), −73.94 (3F, q, 10.1 Hz) ppm; minor: −73.24 (3F, q, 10.1 Hz), −73.75 (3F, q, 10.1 Hz) ppm. ^e^Two partially overlapped sets of signals, ratio 3:1.
